# Waterpipe Nicotine Dependence and Depressive Symptoms among Adolescent Waterpipe and Dual Users

**DOI:** 10.1155/2020/2364571

**Published:** 2020-11-06

**Authors:** Ahmad Abbadi, Jawad Alnahar, Sohaib Zoghoul, Abdullah Bsoul, Salameh Alarood, Abdel-Hameed Al-Mistarehi, Sukaina Alzyoud

**Affiliations:** ^1^World Health Organization, Jordan Country Office, Amman, Jordan; ^2^Hashemite University, Amman, Jordan; ^3^Medical Education Department, Hamad Medical Corporation, Doha, Qatar; ^4^Jordan University of Science and Technology, Irbid, Jordan; ^5^Department of Community and Mental Health, Faculty of Nursing, Hashemite University, Amman, Jordan

## Abstract

**Background:**

Waterpipe nicotine dependence and its association with depressive symptoms and dual usage among adolescents are currently not examined in the literature. Adolescents are a vulnerable population that is susceptible to depression and initiation of tobacco use. We aim, in this novel study, to assess the association between depressive symptoms and waterpipe nicotine dependence among adolescents in Jordan, evaluate the association between waterpipe smoking status (waterpipe smoker vs. dual user) and waterpipe nicotine dependence, and assess the internal validity of the Waterpipe Nicotine Dependence Scale (WNDS).

**Method:**

A cross-sectional study among adolescents of grade 9^th^ to 12^th^ in Jordan was conducted through multistage cluster random sampling. The self-reported Arabic Youth Tobacco Use Composite Measure Questionnaire (YTUCM) was used to collect the surveys that include demographic information, smoking status, and the WNDS to assess waterpipe nicotine dependence and depressive symptoms. Multiple linear regression and the *t*-test were used to analyze the data. *Findings*. One thousand three hundred and three surveys were collected, of which 1082 were included in the study (443 males and 639 females). 64.9% of the sample were nontobacco users, while 20.1% were waterpipe- (WTP-) only smokers, 11.4% were dual users, and 3.7% were cigarettes-only users. After adjusting for weights, 66.6% were nonsmokers, 19.2% were WTP-only smokers, 10.2% were dual users, and 3.9% were cigarettes-only smokers. Using multiple linear regression, depressive symptoms were significantly associated with WTP nicotine dependence (*β* 0.618), upon adjusting for confounders. Furthermore, dual users were associated with higher WTP nicotine dependence (*β* 12.034) compared to WTP-only smokers after adjusting for confounders. Cronbach's alpha for the WNDS was 0.955.

**Conclusions:**

Our study shows that there is a statistically significant association between depressive symptoms and WTP nicotine dependence and higher dependence among dual users compared to WTP-only smokers. The WNDS can be a useful tool to assess WTP nicotine dependence with high internal consistency. However, a longitudinal study is needed to further understand the association and temporality between the depressive symptoms and WTP nicotine dependence. Additionally, research is needed to shorten the WNDS while maintaining high internal consistency and assess the external validity of the WNDS and the short- and long-term consequences of dual usage.

## 1. Introduction

Unlike other regions of the world, where tobacco consumption is decreasing, the East Mediterranean Region (EMR) is increasing in prevalence with projected sustained growth by 2025 [1]. Currently, Jordan has the highest prevalence of male smokers in the region and has the second highest male smokers' prevalence in the world at 70.2% [2]. In 2020, Guardian has reported on a recent unpublished study by the Jordanian Government in collaboration with the World Health Organization (WHO) to show that prevalence of nicotine consumption (from cigarettes, waterpipes, e-cigarettes, and smokeless tobacco) in Jordanian men is estimated to be 82% [3]. According to the Global Youth Tobacco Survey (GYTS), Jordanian youth (13–15 years of age) has the highest level (23.3%) of tobacco smoking in the region [4].

Tobacco is a preventable leading cause of noncommunicable diseases (NCDs) that include cardiovascular diseases, diabetes, cancer, and chronic respiratory diseases [5–7]. The increase in waterpipe usage is a threat to public health [8–11]. Waterpipe (WTP) is one type of tobacco use that can cause diseases similar to those caused by cigarettes use [12–14]. The smell, sounds, and flavors attract youth initiation and are recognized as key characteristics that drive people to use it [8, 15]. Since waterpipes deliver nicotine, dependence can develop as a consequence to consumption [9, 15, 16]. Additionally, exposure to nicotine at an early age of the person's developmental stages in childhood or adolescence could result in brain developmental impairments such as deficiency of working memory [17]. Furthermore, tobacco smoking (cigarettes and waterpipe) is linked to depression and depressive symptoms [18–21], as smokers are more susceptible to develop depressive symptoms compared to nonsmokers [21, 22]. In Jordan, Malak and Khalifeh have reported 73.8% prevalence of depressive symptoms among adolescents aged 12–18 [23], while Ismayilova et al. reported increased depressive symptoms among youth smokers [24].

In Jordan, despite the ratification of the Framework Convention on Tobacco Control (FCTC) and national laws and regulations (i.e., National Public Health Law 47/2008) [25] which prohibit selling tobacco to persons below 18 years of age, evidence shows that tobacco products are accessible to youth and minors [26, 27]. Legally, aforementioned laws not only prohibit selling tobacco to minors, but also forbid allowing minors to enter smoking permitted areas [28, 29]. The Ministry of Health (MOH) decree on tobacco products placement regulations structured the manner in which tobacco products are displayed in all establishments and prohibited opening stores that sell tobacco products close to kindergartens, schools, or healthcare centers [28]. Currently, there are no published studies that directly associate waterpipe nicotine dependence and feeling of depressive symptoms in the EMR region or in Jordan. There are currently two scales that assess WTP nicotine dependence; the first is the Lebanon Waterpipe Dependence Scale (LWDS-11) which was published and internal validity was assessed [30, 31], and the second is the Waterpipe Nicotine Dependence Scale (WNDS) [32]. Our study aims to be one of the first studies to identify the association between depressive symptoms and WTP nicotine dependence among youth tobacco smokers in Jordan, to evaluate the association between WTP smoking status (WTP smoker vs dual user) and WTP nicotine dependence, and to evaluate the internal consistency of the WNDS.

## 2. Methodology

### 2.1. Design

A descriptive cross-sectional study was conducted to determine patterns, behavior, and frequency of tobacco smoking among adolescents, as well as depressive symptoms and WTP nicotine dependence.

### 2.2. Setting

The study was conducted in Irbid, Jordan's second largest governorate located in the northern part of Jordan. Jordan had a total population of 10,053,000 by the end of 2017, of which 18.6% (1,867,000) lived in the Irbid governorate [33]. It is considered the second largest industrial and most densely populated governorate in the country [33]. This governorate was selected because it represents diversity of adolescents from urban, suburban, semirural, and rural population contexts.

### 2.3. Sampling

With more than 100,000 school students in the governorate educational districts, our sample was recruited using a multistage cluster random sampling. There are nine school districts in Irbid categorized in regard to the type of community as follows: 1 urban, 3 suburban, and 5 rural. A list of all schools in nine districts (representing the clusters) was obtained from the Ministry of Education (MOE) to be used for school selection. Initially, sample size calculation was performed. The sample size calculations used power command on STATA 16.1, with the power set at 0.8 and alpha at 0.05. We specified the sample size to be 1250 based on the budget available, and we expected 20 points difference between WTP-only smokers and dual users on the WNDS with 20 SD using a cluster design. The results indicated the need of 4 clusters with 313 participants in each cluster. Therefore, four school districts representing the governorate areas were randomly selected (1 urban, 2 suburban, and 1 rural). Subsequently, lists of all schools in each of the selected districts were used to randomly choose schools (using random numbers generated by random.org) to be included in data collection. The final sampling process resulted in selecting 7 public schools and 16 private ones. Randomly, classes from these schools were designated with those that met the study criteria invited to participate. The research team aimed to include students from 9^th^ to 12^th^ grades. All students in the selected classes from 9^th^ to 12^th^ grades were eligible to participate. The calculated sample size to the study was documented as 1303 responses. However, due to incontrollable technical issues such as viruses, software, or processer errors with few computers in some sample schools, a number of surveys failed to meet our research standards. After excluding the damaged files and all surveys with missing answers or duplicates, our final sample size included was 1082 completed surveys meeting our research standards. The damaged files happened completely at random, and we could not trace them back to the students as all questionnaires were anonymized.

### 2.4. Procedure

The study protocol was approved in December 2018 by the Institutional Review Board of the Hashemite University (HU) number 4/2/2018/2019 and Ministry of Education. To protect the participants' rights, all schools' principals were contacted beforehand to coordinate with the parents of the children for participation consent. Parents received official letters to directly communicate the research interest and to grant permission to allow their children to participate. The letter also included the principle investigator's name, contact information, and an accessible phone number to call for any questions or concerns. Informed consents were obtained from all participants before initiating study measures. Teachers were not present in the class during data collection to prevent the risk of exposing participants' views, behaviors, and choices in regard to tobacco smoking. All study materials were safely locked in a file cabinet in the lead researcher's office.

Data collection was conducted by the research team and a group of medical students from the International Federation of Medical Students' Associations—Jordan (IFMSA-Jo) who were trained on research methods, data collection, and study protocol by the lead researcher. The study questionnaire was administered electronically using computers. The questionnaire took 30 minutes on average to be filled out by the students, with few students requiring modifications due to personal difficulties. On such instances, students were offered additional time to complete their responses. A few others with reading/learning difficulties were supported by the research team by privately reading the questions loud to them without any interpretation of the meaning of the questions.

To address the potential risk due to the sensitivity of the research topic, the questionnaire was anonymized insuring that the provided demographic information does not allow for identity identification. All possible and available resources to address tobacco use in the community were acknowledged before the initiation of the study and were communicated efficiently to the participants, so they can identify and answer the questions related to tobacco products (cigarettes and waterpipes) during the survey. The research team handed out flyers to the participants focusing on harmful health effects of smoking and available smoking caseation resources after data collection. All survey responses were uploaded to a secure drive folder and accessed only by the lead researcher to protect the data.

### 2.5. Measures

The Arabic Youth Tobacco Use Composite Measure Questionnaire was utilized to collect the data, and it was designed as a single measure to assess the pattern of tobacco smoking among youth [34, 35]. The questionnaire consisted of four sections: (a) demographics which includes age, grade, father/mother job, and academic achievements, (b) tobacco smoking status asking about applicable history of smoking habits and waterpipe smoking habits, (c) a depression symptoms scale [21], which includes 6 items assessing the presence of depression, and (d) the Waterpipe Nicotine Dependence Scale (WNDS) which measures the level of nicotine dependence among waterpipe smokers. Response options in the questionnaire varied based on the construct and items measuring that construct including Likert-type responses, yes/no responses, fill in the blank, and multiple-choice questions. The questions of the father's and mother's profession, current employment, and number of family members living in the same household were used to evaluate socioeconomic status. The parents' professions are fill in the blank questions that were later on categorized based on the stated profession as blue collar, white collar, and housemaker. When answering the father's and mother's job, we noticed that the students reported that the parent was deceased in place of the profession, which was included as a new value in our analysis in addition to the predefined values (blue collar, white collar, and housemaker).

The Waterpipe Nicotine Dependence Scale (WNDS) had 36 questions, with 5 response options ranging from (1) not at all true to (5) completely true [32]. Our questionnaire included an additional question “When I have the urge to smoke waterpipes in a place or time that is not possible or allowed, I smoke a cigarette instead,” making the total scale 37 questions. The symptomology increases as the score increases. The responses to each question were summed in one variable, resulting in a scale ranging from 37 to 185, with 111 being the midpoint of the scale. The internal consistency will be assessed in our study.

A similar approach was adopted with the Depressive Symptoms Scale, which had 6 questions, with 4 answer options ranging from (1) rarely or none of the time to (5) most or all of the time [21, 35]. The answers were summed into one variable resulting in a scale ranging from 6 to 24, with 15 being the midpoint of the scale. The questions included in the scale are from the CES-D scale; questions 5, 14, and 17–20 [36]. The brief scale was utilized and assessed previously in similar studies [21, 35]. Generally, the higher the score, the higher the symptomology.

Smoking status variable was the result of combining the answers of the questions: “Do you smoke waterpipe?” and “Do you smoke cigarettes?” Those who have answered yes to both were labelled dual users, while those who answered no to both were labelled nonsmokers. Those who have answered yes to “Do you smoke waterpipe?” but no to “Do you smoke cigarettes?” were labelled as waterpipe-only smokers.

### 2.6. Data Analysis

STATA 16.1 was used for data management and analysis. Data were first reviewed to look for any missing data or impossible answers for questions (e.g., string value for a numerical variable). Responses that included missing values, inconsistency, or any sort of error were excluded from the final data analysis. Following the data cleaning, frequency distribution and percentages were reported. An independent *t*-test was utilized to evaluate difference in means between WTP nicotine dependence, depressive symptoms, and number of WTPs smoked per week among WTP smokers and dual users and between the sexes. Multiple linear regression was performed to assess the potential association between depressive symptoms (exposure) and WTP nicotine dependence (outcome). The regression model was adjusted for the cluster design (by districts) and potential confounders: WTP smoking status, age, sex, grade, socioeconomic status, status of smoking for the parents, siblings, and friends (nonsmoker, cigarettes smoker, waterpipe smoker, or dual user), number of waterpipes smoked per week, and feeling a need to smoke waterpipes. The last year's GPA, a potential mediator, is adjusted for to estimate potential direct effect. The model was also adjusted for weights, but could not adjust for the stratification based on school and class as we were not allowed to collect such data by the MOE as this would enable identification of the students. Furthermore, we generated another model to assess the association between WTP smoking status (exposure) and WTP nicotine dependence (outcome) because the previous model adjusted for potential colliders and mediators between these two variables. Potential confounders considered were age, sex, grade, socioeconomic status, and status of smoking for the parents, siblings, and friends (nonsmoker, cigarettes smoker, waterpipe smoker, or dual user). Depressive symptoms due to the potential of reverse causality between both the exposure and the outcome, we will present the results in a separate column w/o adjusting for it. *p* values <0.05 are considered significant.

## 3. Results

Table 1 shows sample characteristics. The final sample consisted of 1082 students from a total of 23 schools with a response rate of 100%. Participants were 443 (40.9%) males and 639 (59.1%) females. However, upon adjusting for weights, females represented 62.3%, while males represented 37.7%. Among all the students sampled, almost one-third were in the 9^th^ grade, while students in the 11^th^ grade represented the least class sampled. When it comes to the country of origin, the majority of the participants were Jordanians. 77.2% of the fathers were currently employed while only 14.3% of the mothers were currently employed.

### 3.1. Tobacco Smoking Behaviour

Results show that the number of total smokers is 33.4%. Smoking waterpipe only is the most common tobacco smoking behaviour among the students followed by dual smoking, while being a cigarette-only smoker was the least common behaviour (19.2%, 10.2%, and 3.9%, respectively). Nontobacco users composed 66.6% of the participants. Participants reported that 22.6% of mothers and 50.1% of fathers were tobacco smokers, while at least 42.7% of the participants had a sibling who is a tobacco smoker. 43.9% of participants reported that they have a tobacco smoker friend.

### 3.2. Depressive Symptoms

The scale scores ranged between 6 (lowest recorded value) and 24 (highest recorded value), with a mean of 9.550 (9.041, 10.058) and a median of 8 (Table 2). Females had statistically higher mean depressive symptoms compared to males, 9.988 (9.258, 10.718) and 8.825 (8.548, 9.102), respectively.  Table 3 shows the results of the *t*-test comparing waterpipe smokers only and dual users with no statistical significance for the difference in means.

### 3.3. Waterpipe Nicotine Dependence

The scale scores ranged between 37 (lowest recorded value) and 185 (highest recorded value), with a mean of 50.336 (43.657, 57.015) and a median of 41 (Table 2). Cronbach's alpha coefficient for the scale was 0.955, and all subscales ranged from 0.953–0.956. Upon removing item 37, the coefficient for the scale drops to 0.953.

Males had a statistically higher waterpipe nicotine dependence mean of 55.336 (51.506, 59.166) than females who had a mean of 47.309 (40.226, 54.393). Furthermore, when comparing dual users and waterpipe-only users, the mean among dual users was statically higher, 77.107 (69.906, 84.309), compared to 63.763 (59.037, 68.489) among waterpipe-only users (Table 3). Additionally, we have tested for the number of waterpipes smoked per week, and males had a statistically higher mean of 1.742 (0.849, 2.636) compared to 0.740 (0.220, 1.259) for females.

### 3.4. Multiple Linear Regression

A significant association was found between depressive symptoms and WTP nicotine dependence when adjusting to relevant confounders.  Table 4 shows the crude *β* of depressive symptoms of 1.127 (0.548, 1.706). However, after adjusting for all relevant confounders, the *β* was 0.618 with 95% CI (0.362, 0.873). Additionally, if the last year's GPA (a potential mediator) is adjusted for, the depressive symptoms *β* drops to 0.572 with 95% CI (0.333, 0.812), though the results are not presented in Table 4.

Table 5 shows the association between WTP smoking status and WTP nicotine dependence when adjusted to relevant confounders. Compared to WTP smokers only, non-WTP smokers had a statistically lower predicted WTP nicotine dependence by −16.151 and 95% CI (−20.084, −12.219), while dual users had a statistically higher predicted WTP nicotine dependence by 12.034 and 95% CI (6.841, 17.227).

## 4. Discussion

To our knowledge, this study is the first study to examine waterpipe nicotine dependence and depressive symptoms among adolescent dual tobacco users. Depressive mood has been linked to higher nicotine dependence among youth who use cigarettes [18, 19] and usage of waterpipe [21], and this novel study highlights a similar pattern with waterpipe and dual users. This topic is of timely importance as waterpipe and dual tobacco use are becoming more common and customarily accepted among youth and women in Jordan [35, 37] and the world [9]. Since depressive symptoms and depressive disorders are showing sustained growth globally [38] and high prevalence among youth in Jordan [23], the increase in waterpipe smoking puts more vulnerable youths at risk of higher nicotine dependence and, thus, increased cost for cessation treatment [39] and lower chances for successful cessation [40].

Our findings are consistent with previous studies [41, 42] where males reported higher rates of dependence and dual usage compared to females. Similarly, other research findings [43] shared that female participants expressed more depressive symptoms in comparison to males. However, our study shows a significant increase in youth tobacco use prevalence compared to previous studies conducted in Jordan [4, 21]. Although waterpipe use is the highest form of tobacco use among participants, an interesting finding is that dual usage was more prevalent than cigarette-only smoking. Furthermore, our research shows a statistically significant increase of WTP nicotine dependence among dual users compared to WTP-only users. This could be contributed to the assumption that when waterpipe smoking is inaccessible, smokers who suffer from nicotine withdrawal symptoms may opt for a cigarette to satisfy their need and addiction for nicotine [44]. Therefore, the addition of the 37^th^ item in the WNDS is justifiable as it tackles this aspect.

Dual tobacco use is an emerging pattern [9, 45] with its health risks inadequately studied. Although, independently, each type is identified as a contributor to several diseases [7], their synergistic effects remain mostly unknown. The increase in dual usage opens speculations for potential triple or poly-usage of tobacco products [46], with Guardian reporting on an unpublished study conducted by the Jordanian Government and WHO that shows that 16.5% of adult Jordanian men use electronic cigarettes/vape products [3]. There is a need for further research to evaluate the prevalence of potential poly-usage among youth.

The internal consistency of the WNDS is high. Although our test shows high internal consistency, the large number of items and the very high score of 0.995 might indicate redundancy in some questions [47]. Further evaluation is necessitated including factor analysis to reduce the number of items in the scale while preserving the high internal consistency. Moreover, evaluation of the external validity of the scale in different settings and countries is needed. However, WNDS is useful in its current form as a measure of WTP nicotine dependence among youth and in the Jordanian context.

In our study, we have controlled for several potential confounders that might affect the association between depressive symptoms and WTP nicotine dependence. Nevertheless, due to the design of the study, we cannot exclude reverse causality between the exposure and outcome, and a longitudinal study is warranted to assess the nature of this association.

Several studies on cigarettes smoking and depression reported higher smoking initiation and tobacco dependence among depressed individuals and higher risk of development of depression among smokers and those highly dependent [22, 48]. The bidirectionality of the association cannot be excluded [22]. Additionally, since the association between depressive symptoms and WTP smoking status can be in the causal pathway to WTP nicotine dependence, they can be potential mediators rather than confounders as assumed in our models.

The high prevalence of tobacco use among adolescents, the addictive nature of nicotine, and the insufficient implementation of FCTC put youth at high risks [49]. It is evident that tobacco control measures, in alignment with FCTC, are effective in decreasing the prevalence and consumption of tobacco products [50]. Presumably, the increase in tobacco use prevalence and emergence of dual usage among youth within the Jordanian context in these data alludes that FCTC regulations are insufficiently executed. Our research highlights the association between depressive symptoms and WTP nicotine dependence. If the prevalence of WTP smoking or dual usage increases, it can represent an obstacle to the local efforts working on mental health in Jordan. Therefore, additional research is warranted on the implementation of the FCTC policies in Jordan, the effectiveness of such implementation, and the reflection of such policies on depression and smoking behaviour.

### 4.1. Limitations

Our study included the use of self-report tools that relied on participants' memory and interpretation without a follow-up method of validation. Our data included only one Jordanian governorate (the second largest), which may limit the tendency to generalize our results to the rest of the country and potentially other EMR countries. Although our use of a program to collect the surveys made it easier to have complete surveys and supported students not to skip questions, we have lost surveys during our data collection due to technical issues. Additionally, we were limited by our budget to conduct a larger scale study, which might have limited the power due to the cluster design. The anonymity of the questionnaire and the effective communication of the study purposes to the students and parents have resulted in 100% recruitment rate, but we could not collect data linking the student to the class or school. This limited our analysis potential of adjusting to the class and school in our analysis when controlling for the cluster, which is a potential measurement bias.

## 5. Conclusions

It is evident from the study that tobacco use prevalence among youth is high. Dual usage is alarming, as it is more prevalent than cigarette smokers in our sample. Additionally, depressive symptoms were found to be significantly associated with WTP nicotine dependence. Compared to WTP-only smokers, dual users showed higher WTP nicotine dependence. Consequently, the demand for longitudinal studies to explore the temporality of the association between depressive symptoms and WTP nicotine dependence is warranted to further understand the association observed in our study. In addition, the WNDS is a useful tool to assess the WTP nicotine dependence with high internal consistency, but can be further modified to decrease the potential redundant questions and length. Our study urgently recommends new policy measures and pragmatic practices to prevent predicaments similar to the situation in Jordan.

## Figures and Tables

**Table 1:** Demographic distribution.

	Unweighted		Weighted
	No.	%	%
*Sex*
Female	639	59.1	62.3
Male	443	40.9	37.7
Total	1,082	100.0	100.0

*Grade*			
9th	355	32.8	34.8
10th	260	24.0	25.7
11th	148	13.7	14.2
12th	319	29.5	25.3
Total	1,082	100.0	100.0

*Age*			
13	51	4.7	5.0
14	251	23.2	23.9
15	259	23.9	25.2
16	276	25.5	24.5
17	204	18.9	17.5
18	41	3.8	3.9
Total	1,082	100.0	100.0

*Nationality*			
Jordanian	1,017	94.0	93.3
Palestinian	18	1.7	1.7
Syrian	44	4.1	4.7
Iraqi	1	0.1	0.1
Non-Arab	2	0.2	0.2
Total	1,082	100.0	100.0

*Father's job*			
Deceased	24	2.2	2.3
Blue collar	554	51.2	51.8
White collar	504	46.6	45.9
Total	1,082	100.0	100.0

*Father currently working*			
No	246	22.7	22.8
Yes	836	77.3	77.2
Total	1,082	100.0	100.0

*Mother's job*			
Deceased	2	0.2	0.1
Blue collar	32	3.0	3.5
White collar	199	18.4	18.9
Housewife	849	78.5	77.5
Total	1,082	100.0	100.0

*Mother currently working*			
No	928	85.8	85.7
Yes	154	14.2	14.3
Total	1,082	100.0	100.0

*GPA of last year*			
Less than 60%	21	1.9	2.3
60–69%	130	12.0	11.3
70–79%	227	21.0	20.2
80–89%	357	33.0	31.2
90% and above	347	32.1	35.0
Total	1,082	100.0	100.0

*Current waterpipe smoking*			
No	742	68.6	70.5
Yes	340	31.4	29.5
Total	1,082	100.0	100.0

*Current cigarettes smoking*			
No	919	84.9	85.8
Yes	163	15.1	14.2
Total	1,082	100.0	100.0
*Smoking behaviour*			
Nonsmoker	702	64.9	66.6
Cigarettes smoker only	40	3.7	3.9
Waterpipe smoker only	217	20.1	19.2
Current dual user	123	11.4	10.2
Total	1,082	100.0	100.0

*Mother or step mother smoking*			
No	834	77.1	77.4
Cigarettes	160	14.8	14.3
Waterpipe	49	4.5	4.5
Dual user	39	3.6	3.8
Total	1,082	100.0	100.0

*Father or step father smoking*			
No	538	49.7	49.9
Cigarettes	401	37.1	36.4
Waterpipe	69	6.4	6.8
Dual user	74	6.8	6.9
Total	1,082	100.0	100.0

*Siblings smoking*			
No	611	56.5	57.3
Cigarettes	227	21.0	20.0
Waterpipe	101	9.3	9.5
Dual user	143	13.2	13.2
Total	1,082	100.0	100.0

*Friends smoking*			
No	566	52.3	56.1
Cigarettes	209	19.3	16.7
Waterpipe	131	12.1	11.4
Dual user	176	16.3	15.8
Total	1,082	100.0	100.0

**Table 2:** Comparison between females and males∗ǂ.

Variable	All *n* = 1082	Female	Male	*p* value
WTP Nicotine Dependence Scale^1^	50.336 (43.657, 57.015)	47.309 (40.226, 54.393)	55.336 (51.506, 59.166)	0.014
Depressive Symptoms Scale^2^	9.550 (9.041, 10.058)	9.988 (9.258, 10.718)	8.825 (8.548, 9.102)	0.034
Waterpipes smoked per week	1.118 (0.420, 1.816)	0.740 (0.220, 1.259)	1.742 (0.849, 2.636)	0.023

^*∗*^Means followed by 95% CI. ǂAdjusted for weights (the total number of observations after weight adjustment is 123,083) and clusters. ^1^WTP Nicotine Dependence Scale ranges from 37 to 185. ^2^Depressive Symptoms Scale ranges from 6 to 24.

**Table 3:** Comparisons between WTP smokers and dual users∗ǂ.

Variable	WTP smokers	Dual users	*p* value
WTP Nicotine Dependence Scale^1^	63.763 (59.037, 68.489)	77.107 (69.906, 84.309)	0.001
Depressive Symptoms Scale^2^	9.914 (9.364, 10.463)	10.593 (8.926, 12.261)	0.175
Waterpipes smoked per week	2.615 (1.586, 3.644)	4.074 (2.022, 6.127)	0.145

^*∗*^Plus-minus values are means ± SD. ǂAdjusted for weights (the total number of observations after weight adjustment is 123,083) and clusters. ^1^WTP Nicotine Dependence Scale ranges from 37 to 185. ^2^Depressive Symptoms Scale ranges from 6 to 24.

**Table 4:** Multiple linear regression results of waterpipe nicotine dependence (dependent) and depressive symptoms (independent).

Variables	Values	(1)	(2)	(3)
Depressive symptoms		1.127^*∗*^ (0.548, 1.706)	0.859^*∗*^ (0.454,1.263)	0.618^*∗∗*^ (0.362, 0.873)
WTP smoking status	Nonsmoker		−19.972^*∗∗*^ (−21.929, −18.016)	−10.544^*∗∗*^ (−14.116, −6.972)
	Dual user		11.907^*∗∗*^ (6.668, 17.145)	8.162 (−1.951, 18.275)
Sex			5.301^*∗*^ (0.790, 9.812)	2.077 (−2.155, 6.309)
Grade			0.160 (−1.986, 2.306)	−0.179 (−1.396, 1.039)
Age			0.281 (−1.727, 2.289)	0.648 (−0.263,1.560)
Father's work	Blue collar		−0.153 (−13.668, 13.361)	1.207 (−12.629, 15.043)
	White collar		3.109 (−9.780, 15.998)	3.938 (−7.688, 15.565)
Father's current employment			2.151 (−1.352, 5.654)	0.991 (−4.309, 6.291)
Mother's work	Blue collar		−5.784 (−12.495, 0.927)	9.608 (−11.601, 30.816)
	White collar		−10.453^*∗*^ (−19.043, −1.863)	6.312 (−19.880, 32.503)
	Housewife		−10.329^*∗*^ (−17.816, −2.843)	7.302 (−18.520, 33.125)
Mother's current employment			−2.927 (−7.792, 1.939)	−1.738 (−8.020, 4.544)
Family members in the household			0.096^*∗*^ (0.036, 0.156)	−0.117 (−0.253, 0.019)
Mother's smoking status	Cigarettes			0.328 (−3.016, 3.672)
	WTP			−3.047 (−11.475, 5.381)
	Dual user			−2.948 (−12.697, 6.801)
Father's smoking status	Cigarettes			−1.127 (−4.261, 2.006)
	WTP			0.253 (−4.952, 5.457)
	Dual user			2.216 (−6.946, 11.379)
Siblings' smoking status	Cigarettes			1.125 (−1.952, 4.201)
	WTP			3.001^*∗*^ (1.184, 4.818)
	Dual user			2.588^*∗*^ (0.425, 4.752)
Friends' smoking status	Cigarettes			0.204 (−7.339, 8.513)
	WTP			4.007 (−1.493, 9.507)
	Dual user			8.045^*∗∗*^ (5.053, 11.037)
WTP smoked per week				1.634 (−0.564, 3.832)
Feels need to smoke WTP				8.861^*∗*^ (3.849, 13.874)
Constant		39.569^*∗∗*^ (33.015, 46.122)	58.938^*∗∗*^ (44.137, 73.740)	33.515 (−7.260, 74.289)

Statistically significant ^*∗*^(*p* < 0.05)^*∗∗*^(*p* < 0.01). The table shows the point estimate, followed by the 95% confidence interval in brackets. WTP smoking status' reference value was WTP-only smoker, and the other categorical variables used the first value reported above as the reference value. The table shows the results of the regression of the dependent variable waterpipe nicotine dependence calculated from the Waterpipe Nicotine Dependence Scale. The independent variable was depressive symptoms calculated from the depressive symptoms scale. The model controlled for potential confounders: waterpipe smoking status (nonsmoker, waterpipe-only smoker, and dual user), sex, age, grade [9–12], father's and mother's work (deceased, blue collar, white collar, and housemaker) and current employment as a proxy for socioeconomic status with the number of family members in the household, status of smoking for the parents, siblings, and friends (nonsmoker, cigarettes smoker, waterpipe smoker, or dual user), number of waterpipes smoked per week, and feeling a need to smoke waterpipes. The first model represents the crude unadjusted coefficient. The second model adjusts for the sex, grade, age, socioeconomic status, and WTP smoking status. The third model adjusts for all potential confounders considered in our study. The models are adjusted for the clusters and weights generated from the study design.

**Table 5:** Multiple linear regression results of waterpipe nicotine dependence (dependent) and waterpipe smoking status (independent).

Variables	Values	(1)	(2)	(3)
WTP smoking status	Nonsmoker	−20.980^*∗∗*^ (−23.697, -18.262)	−16.847^*∗∗*^ (−21.151, −12.543)	−16.151^*∗∗*^ (−20.084, −12.219)
	Dual user	13.343^*∗∗*^ (9.807, 16.879)	12.331^*∗∗*^ (7.818, 16.845)	12.034^*∗∗*^ (6.841, 17.227)
Sex			2.169 (−2.646, 6.983)	3.173 (−2.342, 8.688)
Grade			−0.159 (−1.864, 1.546)	0.156 (−1.884, 2.196)
Age			0.739 (−0.256, 1.733)	0.343 (−0.886, 1.573)
Father's work	Blue collar		0.866 (−14.146, 15.878)	1.415 (−13.701, 16.531)
	White collar		3.854 (−9.909, 17.618)	4.262 (−9.396, 17.920)
Father's current employment			1.428 (−2.468, 5.325)	1.703 (-2.467, 5.872)
Mother's work	Blue collar		−14.172^*∗∗*^ (−15.202, −13.142)	−7.722^*∗∗*^ (−11.739, −3.704)
	White collar		−18.438^*∗∗*^ (−20.462, −16.414)	−11.758^*∗∗*^ (−16.840, −6.677)
	Housewife		−16.800^*∗∗*^ (−22.403, −11.197)	−10.428∗ (−18.616, −2.239)
Mother's current employment			−1.823 (−8.753, 5.106)	−2.344 (−9.828, 5.140)
Family members in the household			0.084^*∗*^ (0.031, 0.137)	0.084^*∗∗*^ (0.041, 0.126)
Mother's smoking status	Cigarettes		2.195^*∗*^ (0.154, 4.235)	1.653 (−0.064, 3.369)
	WTP		−1.309 (−8.329, 5.711)	−1.106 (−8.584, 6.371)
	Dual user		−4.292 (−12.074, 3.489)	−3.561 (−10.755, 3.632)
Father's smoking status	Cigarettes		−0.361 (−3.470, 2.748)	−0.501 (−3.436, 2.435)
	WTP		−0.251 (−5.479, 4.978)	0.069 (−4.710, 4.848)
	Dual user		3.836 (−3.717, 11.390)	3.819 (−4.593, 12.231)
Siblings' smoking status	Cigarettes		1.249 (−0.780, 3.278)	0.971 (−0.715, 2.657)
	WTP		2.279 (−0.276, 4.833)	2.862^*∗*^ (1.172, 4.553)
	Dual user		4.127^*∗*^ (0.947, 7.306)	3.534^*∗*^ (0.868, 6.200)
Friends' smoking status	Cigarettes		0.680 (−6.576, 7.935)	0.879 (−6.649, 8.406)
	WTP		5.908^*∗*^ (1.594, 10.221)	6.011^*∗*^ (1.263, 10.758)
	Dual user		11.718^*∗∗*^ (10.554, 12.883)	11.318^*∗∗*^ (9.630, 13.006)
Depressive symptoms				0.784^*∗∗*^ (0.437, 1.130)
Constant		63.759^*∗∗*^ (59.036, 68.482)	67.983^*∗∗*^ (51.000, 84.966)	53.324^*∗∗*^ (37.455, 69.194)

Statistically significant ^*∗*^(*p* < 0.05)^*∗∗*^(*p* < 0.01). The table shows the point estimate, followed by the 95% confidence interval in brackets. WTP smoking status' reference value was WTP-only smoker, and the other categorical variables used the first value reported above as the reference value. The table shows the results of the regression of the dependent variable waterpipe nicotine dependence calculated from the Waterpipe Nicotine Dependence Scale. The independent variable was WTP smoking status (WTP-only smoking was used as base value). The model controlled for potential confounders: sex, age, grade [9–12], father's and mother's work (deceased, blue collar, white collar, and housemaker) and current employment as a proxy for socioeconomic status with the number of family members in the household, status of smoking for the parents, siblings, and friends (nonsmoker, cigarettes smoker, waterpipe smoker, or dual user), and depressive symptoms. The first model represents the crude unadjusted coefficient. The second model adjusts for all potential confounders except for depressive symptoms. The third model adjusts for all potential confounders considered in our study. The model is adjusted for the clusters and weights generated from the study design.

## Data Availability

A cleaned subdataset is given in Supplementary Materials relating to the findings presented in this paper.

## Supplementary Materials

Supplementary Materials This is the STATA dataset that was used in this paper. For additional information, inquiries or use of the dataset please contact the corresponding author.
